# Protective Effect of *Agaricus brasiliensis* on STZ-Induced Diabetic Neuropathic Pain in Rats

**DOI:** 10.1155/2014/679259

**Published:** 2014-01-12

**Authors:** Weifeng Ji, Haiying Huang, Ji Chao, Wuchao Lu, Jianyou Guo

**Affiliations:** ^1^Department of Orthopedics, Chinese Orthopedics and Traumatology Research Institute, The First Affiliated Hospital of Zhejiang Chinese Medical University, Hangzhou 310006, China; ^2^School of Pharmacy, Henan University of Traditional Chinese Medicine, Zhengzhou 450046, China; ^3^School of Basic Medicine, Fujian Medical University, Fuzhou 350108, China; ^4^Key Laboratory of Mental Health, Institute of Psychology, Chinese Academy of Sciences, Beijing 100101, China

## Abstract

*Objective*. The present investigation examined the neuroprotective effect of *Agaricus brasiliensis* (AbS) against STZ-induced diabetic neuropathic pain in laboratory rats. STZ-induced diabetic rats were administered orally with AbS. Body weight, serum glucose, and behavioral parameters were measured before and at the end of the experiment to see the effect of AbS on these parameters. After 6 weeks of treatments, all animals were sacrificed to study various biochemical parameters. Treatment with AbS 80 mg/kg in diabetic animals showed significant increase in body weight, pain threshold, and paw withdrawal threshold and significant decrease in serum glucose, LPO and NO level, Na-K-ATPase level, and TNF-**α** and IL-1**β** level as compared to vehicle treated diabetic animals in dose and time dependent manner. AbS can offer pain relief in PDN. This may be of potential benefit in clinical practice for the management of diabetic neuropathy.

## 1. Introduction

Diabetic neuropathy is the most common of secondary complications associated with diabetes mellitus and is characterized by the slowing of nerve conduction velocity, elevated pain, sensory loss, and nerve fiber degeneration. Diabetes induced neuropathic pain is recognized as one of the most difficult types of pain to treat with conventional analgesics.

Current treatment of peripheral diabetic neuropathy (PDN) involves the use of tricyclic antidepressant, selective serotonin reuptake inhibitors [1], anticonvulsants, opioids and antioxidant protein kinase C inhibitors, COX-2 inhibitors [2], nonsteroidal anti-inflammatory drugs as mild analgesics, and so on. However, these therapies provide relief only to a fraction of patients and their side effect profiles limit their use [3, 4]. Thus, there is a need for new therapeutic interventions targeting primary mechanisms resulting in nerve damage in PDN. Recently, from the point of view of “self-medication” or “preventive medicine,” several dietary supplements are used in the prevention of lifestyle related diseases including diabetes.

Mushrooms and primarily basidiomycetous fungi are valuable foods that are low in calories and high in minerals, essential amino acids, vitamins, and fibers [5]. Some of them produce substances with potential medical effect and are called medicinal mushrooms [6]. The basidiomycete *Agaricus brasiliensis* (AbS) is native to Brazil and is widely grown in Japan because of its medicinal properties. It is widely used for nonprescript, medicinal purposes, both as an edible mushroom and in the form of extracts. AbS has traditionally been used for the prevention of a range of diseases, including cancer, hepatitis, atherosclerosis, hypercholesterolemia, diabetes, and dermatitis [7–9].

However, the role of AbS in diabetic complications has not been investigated. The aim of the present investigation was to evaluate the neuroprotective effect of AbS against STZ-induced diabetic neuropathic pain in laboratory rats by assessing various behavioral and biochemical parameters.

## 2. Material and Methods

### 2.1. Animals

Healthy male adult Wistar rats (2 months old and weighing 225 ± 25 g) were used in the study. This study was performed in accordance with the Guide for the Care and Use of Laboratory Animals. Care was taken to minimize discomfort, distress, and pain to the animals.

### 2.2. Chemicals

Streptozotocin was purchased from Sigma (USA) and was dissolved in 0.1 N citrate buffers.

### 2.3. Fermented Mushroom of AbS Extract

The fermented mushroom of AbS was produced by the way to produce *Coprinus comatus *[10]. The aqueous extraction was performed by adding 100 mL boiling water to 10 g air-dried mycelium. The infusion stood at room temperature for 30 minutes. After cooling and filtration, the extract was frozen and concentrated by lyophilization for five days overnight, in order to obtain the AbS (0.68 g).

### 2.4. Induction and Assessment of Diabetes in Rats

Experimental diabetes was induced by a single intraperitoneal (i.p.) injection of STZ (50 mg kg^−1^) freshly dissolved in citrate buffer pH. Serum glucose level was assessed by using enzymatic glucose oxidase peroxidase commercially available kit method, 72 h after STZ induction. Only rats with blood glucose concentration more than 240 mg/dL were considered diabetic and used for the study. Body weight and serum glucose were measured before and at the end of the experiment to see the effect of pharmacological interventions on these parameters. The body weights of the mice were measured every two weeks.

### 2.5. Treatment Schedule and Experimental Protocol

Forty hyperglycemic rats were selected and allocated equally into 4 groups. From then on, the 4 groups of hyperglycemic rats were administered orally (gavage) with saline, AbS (20 mg/kg/d), AbS (40 mg/kg/d), and AbS (80 mg/kg/d), respectively. AbS was dissolved in the same amount of saline. The other 10 normal rats were administered orally with the saline and used as the control group.

Body weight of all animals was recorded on the 0, 2nd, 4th, and 6th weeks of treatment. Blood of all animals was collected through retro-orbital route initially and on the 6th week of treatment to measure the serum glucose levels.

### 2.6. Behavioral Tests

Development of neuropathy was assessed in control and diabetic animals from all groups by evaluation of pain thresholds on the 0, 2nd, 4th, and 6th weeks of respective treatment by assessment of thermal/mechanical hyperalgesia and thermal allodynia [11–13].

### 2.7. Biochemical Assessment

All animals were sacrificed at the end of the study; that is, 6th week and sciatic nerves were rapidly removed and weighed. Tissue homogenates were prepared with 0.1 M Tris-HCl buffer (pH7.4) and supernatant of homogenates was employed to estimate superoxide dismutase (SOD) [14], lipid peroxidation (LPO) [15], nitric oxide (NO content) [16], and membrane bound inorganic phosphate (Na+K+ATPase) [17]. TNF-*α* and IL-1*β* concentrations were quantified by ELISA (Neobioscience, China). According to the manufacturer's instructions, the absorbance (A) was detected at 450 nm (A450). The content of each sample was obtained according to the standard curve.

### 2.8. Acute Toxicity Study

The AbS was administered at a dose of 2000 mg/kg orally to ten healthy adult female Wistar rats. Animals were observed individually for the first four hours after dosing for the presence of any clinical signs, such as changes in skin fur, lacrimation, salivation, piloerection, diarrhea, and mortality. The gross behaviors were observed. Surviving animals were observed for outcomes for a period of 24 hours. The animals were kept under supervision up to 14 days for any sign of toxicity or mortality (OECD Guideline, 2000).

### 2.9. Statistical Analysis

All data were analyzed by a one-way analysis of variance, and the differences between means were established by Duncan's multiple-range test. The data are shown as the mean ± SEM. The significant level of 5% (*P* < 0.05) was used as the minimum acceptable probability for the difference between the means.

## 3. Results

### 3.1. Effect of AbS on Body Weight and Blood Glucose Levels

**Figure 1:**
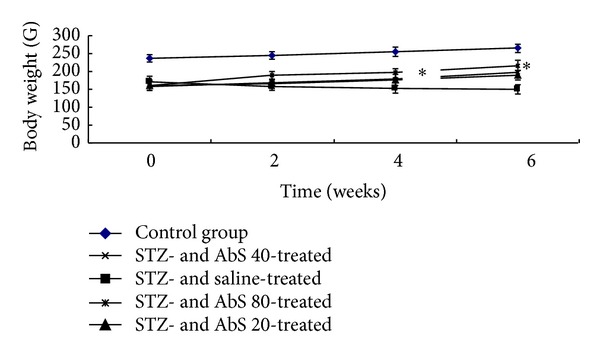
Effect of AbS treatment on body weight. Results are expressed as mean ± SEM (*n* = 6). The data was analysed using and one-way analysis of variance (ANOVA) followed by Dunnett's test. *P < 0.05 versus diabetic group.

The STZ-treated animals had significantly reduced body weight than the control rats. The average blood glucose level of the STZ-treated animals was significantly higher as compared to the control animals. Treatment with AbS 80 mg/kg in diabetic animals showed significant increase in body weight (Figure 1) and significant decrease in serum glucose (Table 1) as compared to vehicle treated diabetic animals in dose and time dependent manner.

### 3.2. Effect of AbS Treatment on Thermal Hyperalgesia

**Figure 2:**
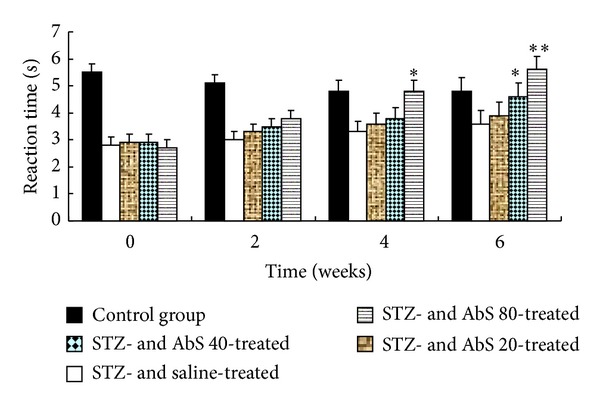
Effect of AbS treatment on tail withdrawal latency. Results are expressed as Mean ± SEM (*n* = 6). The data was analysed using and one-way analysis of variance (ANOVA) followed by Dunnett's test. *P < 0.05 versus diabetic group, **P < 0.01 versus diabetic group.

The nociceptive threshold was significantly lower in diabetic rats as compared with control animals tested in the tail immersion (Figure 2). Treatment of diabetic rats with AbS 80 mg/kg induced a statistically significant increase in pain threshold after four weeks of treatment, which was further increased after six weeks of treatment in dose dependant manner. The AbS 40 mg/kg treatment showed significant increase in pain threshold level as compared to diabetic control only after six weeks of treatment.

### 3.3. Effect of AbS Treatment on Mechanical Hyperalgesia

**Figure 3:**
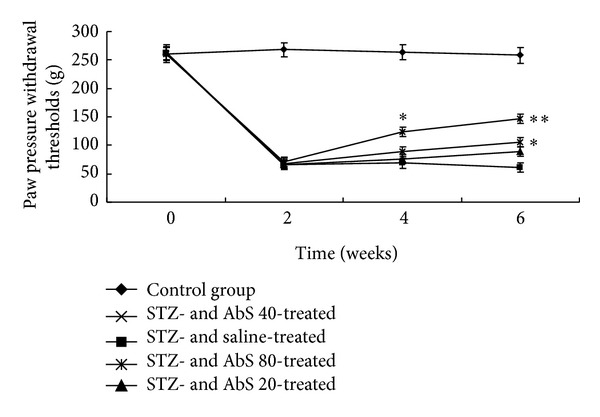
Effect of AbS treatment on paw withdrawal threshold. Results are expressed as mean ± SEM (*n* = 6). The data was analysed using and one-way analysis of variance (ANOVA) followed by Dunnett's test. *P < 0.05 versus diabetic group, **P < 0.01 versus diabetic group.

There was no significant change in the mean paw withdrawal threshold of control rats during the time period of 6 weeks. A significant decrease in (*P* < 0.05) mean paw withdrawal threshold was produced in the diabetic rats (66.21 ± 6.40 g) after 2 weeks of STZ injection as compared to control animals (268.11 ± 14.90 g). In rats receiving the treatment of AbS (40 and 80 mg/kg), mean paw withdrawal threshold was significantly and dose dependently increased (105.50 ± 11.00 and 146.23 ± 22.00 g resp.) compared to diabetic control rats (Figure 3).

### 3.4. Effect of AbS Treatment on Thermal Allodynia

**Figure 4:**
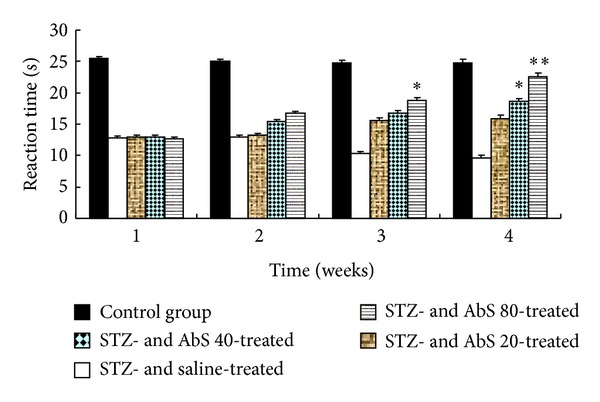
Effect of AbS treatment on paw withdrawal latency in warm plate test. Results are expressed as mean ± SEM (*n* = 6). The data was analysed using and one-way analysis of variance (ANOVA) followed by Dunnett's test. *P < 0.05 versus diabetic group, **P < 0.01 versus diabetic group.

Marked thermal allodynia was observed in the diabetic animals as evidenced by a reduction in the pain thresholds compared to control animals (Figure 4). Treatment of diabetic rats with AbS induced a significant increase in pain threshold compared to diabetic animals after six weeks of treatment in dose dependant manner.

### 3.5. Effect of AbS Treatment Lipid Peroxide Profile and Nitrosative Stress

After 6 weeks of STZ injection, LPO and NO level in diabetic rats was significantly increased as compared to control rats. The LPO level in AbS 80-treated rats was decreased (6.11 ± 0.40 nM/mg of protein, *P* < 0.01) significantly and dose dependently compared to diabetic rats. At the same time, the NO level in AbS 80-treated rats was significantly and dose dependently decreased (169.10 ± 11.21 *μ*g/mL, *P* < 0.05) as compared to diabetic rats (Table 2).

### 3.6. Effect of AbS Treatment on Superoxide Dismutase Profile and Membrane Bound Inorganic Phosphate

Intraperitoneal administration of STZ resulted in significant decrease (*P* < 0.05) in SOD and Na-K-ATPase level in diabetic rats compared to control rats. SOD level in AbS-80 treated rats was increased (20.80 ± 0.63 U/mg of protein, *P* < 0.05) significantly and dose dependently as compared to diabetic rats. At the same time, the Na-K-ATPase level in AbS 80-treated rats was significantly and dose dependently pendently increased (9.11 ± 0.40 *μ*mol/mg of protein, *P* < 0.05) as compared to diabetic rats (Table 3).

### 3.7. Effect of AbS Treatment on TNF-*α* and IL-1*β* Level

As shown in Table 4, the levels of TNF-*α* and IL-1*β* were significantly increased after STZ injection. AbS 80 suppressed STZ-induced TNF-*α* and IL-1*β* production (*P* < 0.05). Moreover, the TNF-*α* and IL-1*β* level in AbS treated rats was dose dependently decreased.

### 3.8. Toxicity Profile

All the animals did not show any sign of toxicity or mortality in the first four hours after dosing and thereafter up to the next 14 days.

## 4. Discussion

Diabetic neuropathy is characterized by clinical features like allodynia and hyperalgesia due to elevated nociceptive response. Similar symptoms are exhibited by STZ-induced diabetic animals [18]. STZ is an antibiotic extracted from *Streptomyces acromogenes* and is diabetogenic due to a selective cytotoxic action upon pancreatic *β* cell [19]. STZ injected rats exhibit clinicopathological features including biochemical, oxidative, and metabolic changes which also presented in humans [20].

In the present investigation, loss in body weight was halted in AbS treated animals when compared with STZ-induced diabetic animals. It indicates that the improvement on body weight may be attributed to the improvement on metabolic dysfunction in diabetic rats. The hypoglycemic effect of AbS was significant exhibiting observation similar to that in earlier reports [21].

It has been reported earlier that STZ-induced diabetic neuropathic pain is characterized by hyperalgesia and allodynia [22, 23] and was also found in the present study following STZ injection. A decrease in pain threshold was observed in STZ-diabetic rats using a mildly noxious stimulus such as mechanical force. Moreover, there was also a similar increase in thermal hyperalgesic activity in diabetic rats as compared with normal rats when subjected to thermal stimuli. In the present investigation, we found that administration of AbS dose dependently reversed STZ-induced thermal hyperalgesia and mechanical allodynia. However, the mechanism of antihyperalgesic and antiallodynic effect of AbS on STZ-induced PDN needs to be further studied.

In diabetes, pain threshold of the neurons is reduced due to oxidative stress generated by free radicals such as super oxide dismutase, hydroxyl radical, and peroxynitrite which impair blood supply to the neurons leading to impaired neuronal function and hypoxia [24, 25]. These conditions were investigated by assessing the biochemical markers like SOD, LPO, Na-K-ATPase, and NO.

We observed a significant increase in LPO and reduction in endogenous antioxidant enzymes like SOD and Na-K-ATPase activity in sciatic nerves of diabetic rats. Treatment with AbS for six weeks restored above mentioned biochemical parameters in diabetic rats in dose dependent manner.

Nitric oxide (NO) is an unconventional intracellular messenger playing a vital role in various pathological and physiological processes. A localized increase in NO level leads to the formation of peroxynitrite by reacting with superoxide anions which causes rapid protein nitration or nitrosylation, lipid peroxidation, DNA damage, and cell death and which intern contribute to elevated pain [26]. NO, an indicator of nitrosative stress, was measured and found to be increased in the STZ-diabetic rats. We observed that AbS treatments dose dependently attenuated nitrite level in STZ-diabetic rats that exhibited thermal hyperanalgaesia and mechanical allodynia.

Evidence from both animal models and humans indicates that systemic inflammation is involved in the pathophysiological processes of diabetes [27]. Moreover, TNF-*α* has been reported to initiate the release of other inflammatory cytokines including IL-1*β* and IL-2 that are responsible for causing neuropathic pain [28, 29]. Uses of agents that suppress cytokine elevation have been advocated to be used to treat diabetic complication. The present investigation demonstrates that AbS was able to dose dependently reduce the population of cytokine. Therefore, AbS may exhibit its neuroprotective effect by downregulation of cytokine including TNF-*α* and IL-1*β*, the important mediators of neuropathic pain.

## 5. Conclusion

These results suggest that AbS attenuated STZ-induced neuropathic pain behaviours by inhibiting oxidative, nitrosative stress, cytokines activation, and hypoglycemic effect of Abs. This may be of potential benefit in clinical practice for the management of diabetic neuropathy.

## Figures and Tables

**Table 1:** Effect of AbS and other treatments on blood glucose levels in STZ-hyperglycemic rats.

Different groups	Blood glucose (mg/dL)
STZ- and saline-treated	310.20 ± 2.111
STZ- and AbS 80-treated	140.56 ± 2.150**
STZ- and AbS 40-treated	196.60 ± 3.226*
STZ- and AbS 20-treated	238.93 ± 2.836
Control group	105.94 ± 1.223

Values are shown as means ± SEM. *P < 0.05 versus diabetic group, **P < 0.01 versus diabetic group.

**Table 2:** Effect of AbS and other treatments on LPO and NO level.

Different groups	LPO (nM/mg of protein)	NO (*μ*g/mL)
STZ- and saline-treated	12.29 ± 0.33	288.30 ± 6.25
STZ- and AbS 80-treated	6.11 ± 0.40**	169.10 ± 11.21*
STZ- and AbS 40-treated	8.22 ± 0.32*	223.21 ± 22.71
STZ- and AbS 20-treated	10.00 ± 0.12	242.20 ± 18.70
Control group	2.20 ± 0.21	108.22 ± 8.13

Values are shown as means ± SEM. *P < 0.05 versus diabetic group, **P < 0.01 versus diabetic group.

**Table 3:** Effect of AbS and other treatments on SOD and Na+K+ATPase level.

Different groups	SOD (U/mg of protein)	Na+K+ATPase (*μ*mol/mg of protein)
STZ- and saline-treated	5.80 ± 0.66	2.29 ± 0.30
STZ- and AbS 80-treated	20.80 ± 0.63*	9.11 ± 0.40*
STZ- and AbS 40-treated	11.60 ± 0.22	6.22 ± 0.32
STZ- and AbS 20-treated	8.93 ± 0.36	4.00 ± 0.10
Control group	29.66 ± 0.80	12.20 ± 1.20

Values are shown as means ± SEM. *P < 0.05 versus diabetic group.

**Table 4:** Effect of AbS and other treatments on TNF-*α* level.

Different groups	TNF-*α* (pg/mL)	IL-1*β* (pg/mL)
STZ- and saline-treated	128.29 ± 10.33	130.29 ± 22.30
STZ- and AbS 80-treated	76.11 ± 10.40**	56.55 ± 9.22**
STZ- and AbS 40-treated	108.22 ± 10.30*	94.11 ± 8.40*
STZ- and AbS 20-treated	111.0 0 ± 10.10	116.10 ± 22.41**
Control group	52.23 ± 4. 20	22.20 ± 2. 20

Values are shown as means ± SEM. *P < 0.05 versus diabetic group, **P < 0.01 versus diabetic group.
